# Analysis of mitochondrial membrane potential, ROS, and calcium

**DOI:** 10.1016/j.mocell.2025.100238

**Published:** 2025-06-16

**Authors:** Hye-Kyung Park, Byoung Heon Kang

**Affiliations:** Department of Biological Sciences, Ulsan National Institutes of Science and Technology (UNIST), Ulsan 44919, South Korea

**Keywords:** Calcium, Membrane potential, Mitochondria, MitoSOX, Rhod-2AM

## Abstract

Mitochondria play a central role in cellular energy metabolism and signaling, and their dysfunction is associated with a wide range of diseases. Therefore, assessing mitochondrial function is essential for understanding their role in various cellular processes and disease progression. Here, we describe the principles and methodologies for analyzing mitochondrial membrane potential, reactive oxygen species, and calcium levels using the fluorescent probes tetramethylrhodamine methyl ester, MitoSOX, and Rhod-2AM, respectively. This work provides a practical guide for researchers investigating mitochondrial function under physiological and pathological conditions.

## INTRODUCTION

Mitochondria are central hubs of cellular energy metabolism and signaling pathways, and their dysregulation is frequently observed in major human diseases, including cancers, metabolic disorders, and neurodegeneration (Harrington et al., 2023, Li et al., 2024). Assessing changes in the mitochondrial activity is critical for understanding the onset and progression of these diseases (Rey et al., 2022, Wallace, 2012, Yang, 2025).

Mitochondrial energy metabolism relies on the transfer of electrons, originating from NADH produced in the tricarboxylic acid cycle, to oxygen through the electron transport chain complexes (Complexes I-IV), resulting in the generation of the mitochondrial membrane potential (ΔΨ_m_) (Lee and Yoon, 2024). During this process, reactive oxygen species (ROS) are produced, serving as critical signaling molecules under physiological conditions and acting as damaging agents under oxidative stress (Sies and Jones, 2020). This metabolic activity is further modulated by mitochondrial calcium levels, which are dynamically controlled by cellular signaling pathways. Changes in mitochondrial metabolism, ROS production, and calcium buffering capacity rewire cellular signaling pathways beyond the mitochondria.

Therefore, the analysis of ΔΨ_m_, ROS, and calcium levels provides a rapid and informative approach to assess overall mitochondrial function, offering critical insights into cellular energy metabolism and signaling pathways. Here, we present analysis protocols using the fluorescent probes tetramethylrhodamine methyl ester (TMRM), MitoSOX, and Rhod-2AM to assess ΔΨ_m_, mitochondrial ROS, and mitochondrial calcium levels, respectively.

## FLUORESCENCE-BASED METHODS FOR MITOCHONDRIAL FUNCTION ANALYSIS

Mitochondrial membrane potential (ΔΨ_m_) can be assessed using cationic fluorescent rhodamine derivatives, such as TMRM (Scaduto and Grotyohann, 1999). TMRM readily crosses the inner mitochondrial membrane due to its lipophilic nature and accumulates in the negatively charged mitochondrial matrix as a result of its cationic properties (Gottschalk et al., 2024). Since TMRM reversibly accumulates in mitochondria in proportion to the membrane potential (ΔΨ), ΔΨ_m_ can be quantitatively estimated from fluorescence intensity once passive diffusion reaches equilibrium, according to the following Nernst equation (Ehrenberg et al., 1988, Farkas et al., 1989).

$$\Delta \Psi =\frac{\mathrm{RT}}{\mathrm{zF}}\ln\left(\frac{{\left[\mathrm{TMRM}\right]}_{\mathrm{outside}}}{{\left[\mathrm{TMRM}\right]}_{\mathrm{inside}}}\right)≅25.7\ln\left(\frac{{\left[\mathrm{TMRM}\right]}_{\mathrm{outside}}}{{\left[\mathrm{TMRM}\right]}_{\mathrm{inside}}}\right)\;(\mathrm{mV})$$

Because TMRM fluorescence intensity is proportional to its concentration, ΔΨ can be estimated by substituting fluorescence intensities for TMRM concentrations in the equation.

MitoSOX is a conjugate of a fluorogenic probe, dihydroethidium, for the detection of superoxide radicals (O₂⁻) and triphenylphosphonium for mitochondrial targeting. The triphenylphosphonium moiety enables MitoSOX to accumulate within mitochondria due to its lipophilic and cationic properties, similar to TMRM (Chung and Duchen, 2022, Smith et al., 2012). Upon oxidation by superoxide in the mitochondrial matrix, MitoSOX is converted to 2-hydroxyethidium, a fluorescent product that enables the specific detection of mitochondrial superoxide levels (Murphy et al., 2022). Impaired mitochondrial function can lead to reduced MitoSOX accumulation due to the loss of membrane potential, and the oxidized MitoSOX products may redistribute within the cell and bind to nuclear DNA, thereby amplifying the fluorescence signal independently of superoxide levels. Therefore, ROS measurement using MitoSOX is most appropriate for comparative analysis rather than absolute quantification. MitoSOX is available as red and green fluorescent probes, facilitating versatile detection of mitochondrial ROS.

Rhod-2AM is a cell-permeable acetoxymethyl ester form of the Ca²⁺-sensitive fluorescent dye Rhod-2. Once inside the cell, it is hydrolyzed by intracellular esterases to yield cell-impermeable Rhod-2, which accumulates in mitochondria due to its positive charge (Davidson and Duchen, 2012). Upon binding to Ca²⁺, Rhod-2 exhibits increased fluorescence, allowing for the monitoring of mitochondrial calcium levels. However, variability in mitochondrial Rhod-2 accumulation due to its dependence on the ΔΨ_m_, as well as the nonlinear relationship between calcium concentration and fluorescence intensity, limits its use to comparative analysis rather than absolute quantification (Table 1).

**Table 1:** Summary of fluorescence probes for mitochondrial imaging

Probe	Target parameter	Sensitivity and responsiveness	Specificity	Notes
TMRM	ΔΨ_m_	High sensitivity with rapid response to changes in ΔΨ_m_	High for polarized mitochondria	– Use <200 nM to avoid fluorescence quenching at high concentration – Comparison before and after FCCP treatment is essential
MitoSOX	Mitochondrial superoxide	Moderate to high; optimal with 10-30 min staining	Moderate; other ROS may interfere with signal	– Oxidation products may diffuse into the cytoplasm or nucleus – Suitable for relative, not absolute, quantification
Rhod-2AM	Mitochondrial calcium	Moderate; signal develops within 30-60 min staining	Moderate; partial cytosolic signal possible	– Possible incomplete mitochondrial localization; mitochondrial marker costaining is recommended – Esterase activity varies by cell type and affects staining – Suitable for relative quantification

ROS, reactive oxygen species; TMRM, tetramethylrhodamine methyl ester.

Collectively, TMRM, MitoSOX, and Rhod-2AM are suitable for a variety of applications, including live-cell fluorescence imaging, flow cytometry, and microplate-based assays. Among these, imaging is particularly useful, as it enables simultaneous assessment of both functional characteristics and structural features of mitochondria. Accordingly, this study focuses on imaging-based methods to describe the experimental approaches. Representative human and mouse cell lines of diverse tissue origin (HeLa, MDA-MB-231, and B16F10) were used to demonstrate the broad applicability of these imaging protocols.

## LIVE-CELL STAINING PROTOCOLS FOR TMRM, MITOSOX, AND RHOD-2AM

**Fig. 1:**
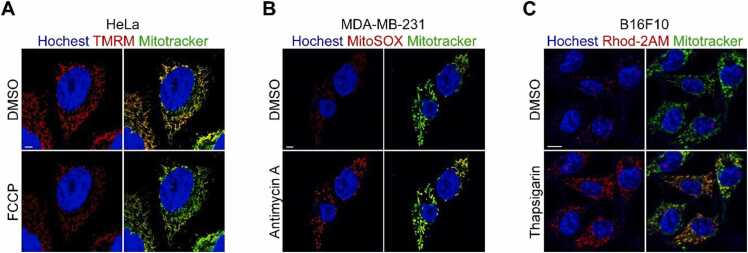
Live-cell imaging using (A) TMRM, (B) MitoSOX, and (C) Rhod-2AM.

1. **Preparation of reagents**: TMRM, MitoSOX, and Rhod-2AM are typically provided dissolved in DMSO, prepared as 1 mM stock solutions, and diluted to the desired working concentration immediately before use.
2. **Staining**: Cells are first washed to remove residual culture medium, either with PBS for TMRM/MitoSOX or with Krebs-Ringer-Hepes (KRH) buffer for Rhod-2AM. Fresh medium for TMRM/MitoSOX and KRH buffer for Rhod-2AM containing the respective dye is then added: 50 to 100 nM TMRM, 5 to 10 μM MitoSOX, or 1 to 5 μM Rhod-2AM. The cells are incubated with TMRM and MitoSOX for 10 to 30 minutes, with Rhod-2AM for 30 to 60 minutes at 37°C in a 5% CO₂ incubator. The staining time and probe concentration may require optimization depending on the cell type. KRH buffer typically contains 140 mM NaCl, 5 mM KCl, 2 mM CaCl₂, 1 mM MgCl₂, 10 mM HEPES (pH 7.4), and 5 mM glucose, with the concentrations of Ca²⁺ and glucose adjustable to match specific culture conditions.
3. **Washing**: Cells are washed 2 to 3 times to remove excess dye using PBS (for TMRM/MitoSOX) or KRH buffer/PBS (for Rhod-2AM). Following washing, cells should be maintained in an appropriate solution: culture medium containing 10 nM TMRM to prevent dye loss; fresh medium for MitoSOX; fresh KRH buffer for Rhod-2AM. For flow cytometry analysis, it is advisable to use PBS in place of the medium or KRH buffer.
4. **Fluorescence analysis**: Fluorescence can be measured using a fluorescence microscope or flow cytometry, with peak excitation/emission wavelengths of 552/574 nm for TMRM, 510/580 nm (Red) or 488/580 nm (Green) for MitoSOX, and 550/590 nm for Rhod-2AM. Costaining with Hoechst (nucleus) and MitoTracker (mitochondria) allows for simultaneous visualization of nuclear and mitochondrial localization and morphology via fluorescence microscopy (Fig. 1 and Table 2).

**Table 2:** Common technical limitations and recommended solutions

Limitation	Cause	Recommended solutions
Nonspecific signals	1. Excessive staining 2. Insufficient washing 3. Use of multiple probes and spectral overlap between probes	1. Reduce probe concentration and/or staining time 2. Perform additional washes 3. Inspect excitation and emission wavelength; adjust imaging filters and setting
Weak signals	Photobleaching due to prolonged light exposure	Minimize exposure time and lower the laser power; handle samples in the dark
Lack of signal validation	1. Probe accumulation in nonmitochondrial compartments 2. No control for signal specificity	1. Confirm mitochondrial localization using MitoTracker 2. Validate probe responsiveness by treating cells with antimycin A (↑[ROS]_m_), MitoTEMPO (↓[ROS]_m_), FCCP (↓ΔΨ_m_, ↓[Ca^2+^]_m_)

ROS, reactive oxygen species.
5. **Control suggestion:** To validate TMRM and Rhod-2AM signals, ΔΨ_m_ can be depolarized using appropriate stressors such as proton ionophores like FCCP, which also induce mitochondrial calcium release. For MitoSOX, ROS can be reduced using mitochondria-targeted scavengers such as MitoTEMPO; however, certain scavengers, such as MitoQ, are not recommended, as they have been reported to disrupt mitochondrial proteostasis beyond ROS scavenging (Yoon et al., 2021).

HeLa, MDA-MB-231, and B16F10 cells were stained with 100 nM TMRM (A), 5 µM MitoSOX Red (B), and 5 µM Rhod-2AM (C). To perturb mitochondrial function, cells were subsequently treated with FCCP (1 µM), antimycin A (1 µM), or thapsigargin (10 µM). All samples were costained with MitoTracker (100 nM, green; ex/em: 490/516 nm) and Hoechst 33342 (5 µg/mL, blue; ex/em: 350/461 nm). Scale bar = 5 µm.

## CONCLUDING REMARKS

Here, we provide an overview of methods and underlying principles for assessing mitochondrial activities. We hope this serves as a valuable reference for researchers who are not familiar with mitochondrial analyses but require a foundational guide for these techniques to study diverse biological pathways.

## Funding and Support

This work was supported by a 10.13039/501100003725National Research Foundation of Korea (NRF) grant, funded by the Korean government (MSIT) (NRF-2022R1C1C2010351 and RS-2024-00342382).

## Author Contributions

**Byoung Heon Kang:** Writing – review & editing, Writing – original draft. **Hye-Kyung Park:** Writing – review & editing, Writing – original draft.

## Declaration of Competing Interests

The authors declare that they have no known financial interests or personal relationships that could have influenced the work reported in this paper.
